# Human Metapneumovirus Phosphoprotein Independently Drives Phase Separation and Recruits Nucleoprotein to Liquid-Like Bodies

**DOI:** 10.1128/mbio.01099-22

**Published:** 2022-05-10

**Authors:** Kerri Beth Boggs, Kearstin Edmonds, Nicolas Cifuentes-Munoz, Farah El Najjar, Conny Ossandón, McKenna Roe, Chao Wu, Carole L. Moncman, Trevor P. Creamer, Gaya K. Amarasinghe, Daisy W. Leung, Rebecca Ellis Dutch

**Affiliations:** a Department of Molecular and Cellular Biochemistry, University of Kentucky, College of Medicine, Lexington, Kentucky, USA; b Instituto de Ciencias Biomédicas, Facultad de Ciencias de la Salud, Universidad Autónoma de Chile, Santiago, Chile; c Facultad de Ciencias del Mar y Recursos Biológicos, Universidad de Antofagasta, Antofagasta, Chile; d Department of Pathology and Immunology, Washington University School of Medicine, St. Louis, Missouri, USA; e Department of Medicine, Washington University School of Medicine, St. Louis, Missouri, USA; Columbia University Medical College

**Keywords:** HMPV, inclusion bodies, phase separation, pneumovirus

## Abstract

Human metapneumovirus (HMPV) inclusion bodies (IBs) are dynamic structures required for efficient viral replication and transcription. The minimum components needed to form IB-like structures in cells are the nucleoprotein (N) and the tetrameric phosphoprotein (P). HMPV P binds to the following two versions of the N protein in infected cells: N-terminal P residues interact with monomeric N (N^0^) to maintain a pool of protein to encapsidate new RNA and C-terminal P residues interact with oligomeric, RNA-bound N (N-RNA). Recent work on other negative-strand viruses has suggested that IBs are, at least in part, liquid-like phase-separated membraneless organelles. Here, HMPV IBs in infected or transfected cells were shown to possess liquid organelle properties, such as fusion and fission. Recombinant versions of HMPV N and P proteins were purified to analyze the interactions required to drive phase separation *in vitro*. Purified HMPV P was shown to form liquid droplets in isolation. This observation is distinct from other viral systems that also form IBs. Partial removal of nucleic acid from purified P altered phase-separation dynamics, suggesting that nucleic acid interactions play a role in IB formation. HMPV P also recruits monomeric N (N^0^-P) and N-RNA to droplets *in vitro*. These findings suggest that HMPV P may also act as a scaffold protein to mediate multivalent interactions with monomeric and oligomeric N, as well as RNA, to promote phase separation of IBs. Together, these findings highlight an additional layer of regulation in HMPV replication by the viral P and N proteins.

## INTRODUCTION

Human metapneumovirus (HMPV), discovered in 2001, is a leading cause of severe respiratory tract infections in infants, the elderly, and immunocompromised individuals (1). A total of 5% to 20% of hospitalizations from respiratory infections in young children are due to HMPV (2, 3). Symptoms of HMPV infection are similar to respiratory syncytial virus (RSV) and include fever, cough, rhinorrhea, croup, bronchiolitis, pneumonia, and asthma exacerbation (4). HMPV and RSV are members of the *Pneumoviridae* family, a viral family that was created in 2016 and classified within the *Mononegavirales* order (5). Currently, no vaccines or antiviral treatments are approved to treat HMPV infections, so most patients are managed with supportive care (4). The recent discovery of HMPV highlights the need to understand the basic mechanisms of its life cycle. Specifically, analyzing the process of HMPV replication may be crucial for identifying new targets for antiviral development.

Along with the pneumoviruses HMPV and RSV, other relevant human pathogens within the *Mononegavirales* order include Ebola virus, measles virus (MeV), and rabies virus (RABV), which have negative-sense, single-stranded RNA genomes. Although these viruses are classified within different families, they have all been reported to form membraneless cytoplasmic structures within infected cells known as inclusion bodies (IBs) (6–9). For some negative-sense, single-stranded RNA viruses, including HMPV, IBs have been shown to house active viral replication and transcription (10–17). These processes involve several viral proteins, such as the large RNA-dependent RNA-polymerase (L), phosphoprotein (P), and nucleoprotein (N). Further analysis of these structures has shown that RSV, MeV, RABV, and vesicular stomatitis virus (VSV) IBs possess the properties of liquid organelles formed via phase separation (15, 18–21). Phase separation is a physical process by which a homogenous fluid separates into two distinct liquid phases (22). Phase separation plays a role in the formation of a variety of membraneless cellular compartments, such as processing bodies (P-bodies), stress granules, and nucleoli, to concentrate specific proteins and nucleic acids, particularly RNA (23). Properties that define these structures as liquid organelles include the ability to undergo fusion and fission, rapid diffusion of internal contents, and a spherical shape due to surface tension (24). Although phase separation has been shown to play a role in the formation of IBs for some viruses, the physical mechanisms and materials that mediate this process in the viral life cycle are still poorly understood.

For RSV, HMPV, MeV, and RABV, the minimum viral components required to reconstitute IB-like structures in cells are the N protein, which encapsidates the RNA genome, and the P protein, which acts as a cofactor to mediate interactions between N and L (15, 19, 25, 26). VSV also requires the presence of the L protein with the N and P proteins to form IBs (18). These findings suggest that interactions between the N and P proteins regulate phase separation to form IBs as a structural platform for viral replication and transcription. Most studies thus far have focused on cellular experiments to investigate viral IB liquid dynamics, but recent publications on MeV and RSV have shown the importance of utilizing purified protein systems to analyze interactions between the N and P proteins *in vitro* (20, 21). For MeV, the recombinant Escherichia coli-expressed and purified P and monomeric N proteins failed to phase separate independently, but they formed liquid droplets when mixed, similar to the requirements for IB formation observed in cells. Interestingly, when RNA was added to MeV N/P liquid droplets, it was incorporated into the droplets and led to the formation of nucleocapsid-like particles that were detected by electron microscopy (20). *In vitro* experiments with recombinant E. coli-expressed RSV proteins showed that RNA-bound N protein rings and P protein form phase-separated liquid droplets when combined in solution (21). These findings support the model that viral IBs form via phase separation, and this mechanism is highly dependent upon interactions between the N and P proteins. This process may enhance viral replication and transcription for RSV.

The HMPV life cycle begins with the virus attaching and fusing to a target cell to release its ribonucleoprotein into the cytoplasm. The ribonucleoprotein structure protects the genome from host nucleases and detection by host pattern recognition receptors and acts as a template for the L protein. The genome is used to generate capped and polyadenylated viral mRNA transcripts that are translated by the host cell ribosomal machinery. The genome is also replicated to make positive-sense antigenome copies that can then be used to generate more negative-sense genome to package into new virions.

The P protein acts as an adaptor to regulate interactions between the polymerase and RNA template during transcription and replication. It functions as a tetrameric protein, in which the monomers interact through a central oligomerization domain (27, 28). The oligomerization domain is flanked by large intrinsically disordered regions (IDRs) that give HMPV P the ability to interact with a variety of binding partners (27). For instance, the C terminus of the P protein interacts with RNA-bound N protein to chaperone attachment to the polymerase. Additionally, HMPV P maintains a monomeric pool of RNA-free N protein (N^0^) through an interaction involving the HMPV P N terminus with the C-terminal domain of the N protein (29). The monomeric N^0^ protein can then be used for ribonucleoprotein assembly at sites of replication where the polymerase synthesizes nascent RNA (29). HMPV P also recruits the antitermination factor M2-1 to the polymerase during transcription to bind nascent viral mRNA (30). A structural analysis of the HMPV polymerase/P protein complex showed the versatility of P monomer interactions with the polymerase, suggesting that IDRs in the P protein modulate a variety of polymerase functions as well (31). Beyond transcription and replication, HMPV P has been shown to play a role in direct cell-to-cell spread of infection by interacting with actin, or an actin-binding protein, to reorganize the host cell cytoskeleton (32).

This is the first report to analyze phase separation for HMPV IBs. We utilized cellular and purified protein systems to analyze phase separation of HMPV proteins to support the characterization of HMPV IBs as liquid organelles and to determine the interactions required for phase separation. We report that HMPV IBs are liquid-like membraneless structures that rely on N/P protein interactions. Our *in vitro* data show that HMPV N and P undergo phase separation and colocalize within liquid droplets when they are mixed in solution. In contrast to MeV and RSV, the HMPV P protein undergoes phase separation in the absence of other viral protein binding partners *in vitro*, suggesting that the P protein may be the key protein to mediate protein interactions to promote IB formation during infection. Wild-type (WT) RNA-bound N protein rings formed aggregates in solution but incorporated into liquid droplets in the presence of the P protein. These findings suggest for the first time that HMPV P acts as a scaffold protein to support multivalent interactions with HMPV N to promote phase separation and IB formation.

## RESULTS

### HMPV P localizes to liquid-like IBs in transfected and infected cells.

**FIG 1:**
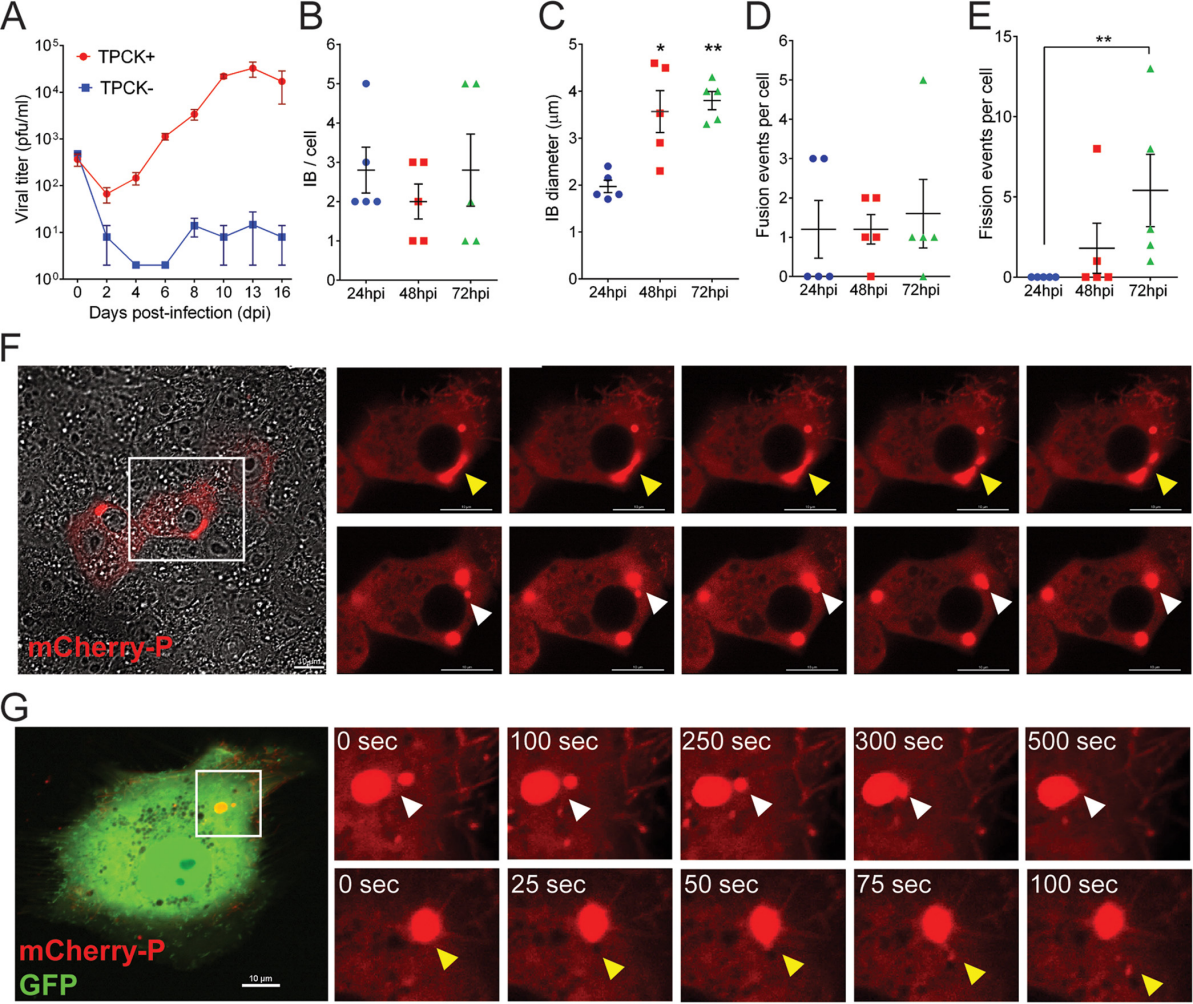
Characterization of a recombinant HMPV-mCherryP virus. (A) Vero cells were infected with an MOI of 0.1, and cells were kept in the absence (TPCK−) or presence (TPCK+) of trypsin until day 16 postinfection. Virus was harvested from the cell supernatants every other day and titrated. Vero cells were infected with HMPV-mCherryP virus using an MOI of 3 to quantify the number of IBs per cell (B) and IB diameter (C) at different times postinfection. Fusion (D) and fission (E) events were counted in Vero cells infected with HMPV-mCherryP at an MOI of 3, during a lapse of 10 min. Images were acquired every 30 sec using a LionHeartFX fluorescence microscope. (F) Time-lapse microscopy of Vero cells infected with HMPV-mCherryP, highlighting fission events (top; yellow arrowheads) and fusion events (bottom; white arrowheads). (G) Vero cells were electroporated with a plasmid encoding mCherryP and subsequently infected with rgHMPV-GFP virus. Forty-eight hours postinfection time-lapse microscopy was performed using a NikonA1 confocal microscope, with images acquired every 25 sec. Fusion (white arrowheads) and fission (yellow arrowheads) events are shown. Statistical analysis was performed using Student’s *t* test. *, *P* < 0.1; **, *P* < 0.01.

During HMPV infection, incoming and newly synthesized ribonucleoproteins concentrate together in the cytoplasm in an actin-dependent manner (11, 33). Eventually, the coalescence of these structures induces the formation of IBs where viral RNA, viral mRNA, P protein, and N protein are detected (11). Inhibition of actin polymerization significantly reduces HMPV genome transcription and replication, suggesting that IB coalescence enhances the efficiency of these processes (11). To gain insights into IB dynamics in HMPV-infected cells, we generated a recombinant virus with an N terminus mCherry-tagged P protein. mCherry-P retained at least 60% of activity in minireplicon assays, while tagging P on its C terminus resulted in deleterious effects (data not shown). To characterize the growth kinetics of the recombinant HMPV-mCherryP virus, Vero cells were infected at a multiplicity of infection (MOI) of 0.1 PFU/cell. Infected cells were maintained in the presence (TPCK+) or absence (TPCK−) of trypsin until 16 days postinfection. Viral titers from supernatants increased until day 10, after which virus growth reached a plateau (Fig. 1A). As expected, HMPV did not grow efficiently in the absence of TPCK trypsin (Fig. 1A). Viral titers for the recombinant HMPV-mCherryP virus were slightly lower than those reported previously for the recombinant JPS02-76EGFP virus (34), but this finding was expected since the mCherryP protein did not retain full replicative activity. It was shown previously that IBs coalesce to a small number of larger structures over the early part of infection and that this process correlates with maximization of replication efficiency (11). In agreement with this result, low numbers of IBs were detected in HMPV-mCherryP infected cells at 24 to 72 h postinfection (hpi) (Fig. 1B). In addition, the size of IBs was shown to nearly double, from 2 μm to almost 4 μm, from 24 to 72 hpi (Fig. 1C), suggesting a maturation of IBs and potentially increased replication during this period. Using live-cell imaging, frequent events of fusion and fission between IBs were observed (Fig. 1D to F). The frequency of fission events per cell increased significantly from 24 to 72 hpi (Fig. 1E), coinciding with the increase in size observed at these hpi. Additionally, incorporation of mCherryP into IBs was observed in mCherryP electroporated-rgHMPV-infected cells (Fig. 1G). Fusion and fission events of the IBs in these transfected-infected cells was observed using live-cell imaging, suggesting that HMPV P has an inherent propensity to be incorporated into IBs. Altogether, our results suggest that as infection progress, HMPV IBs grow and increase in complexity and dynamic behavior.

**FIG 2:**
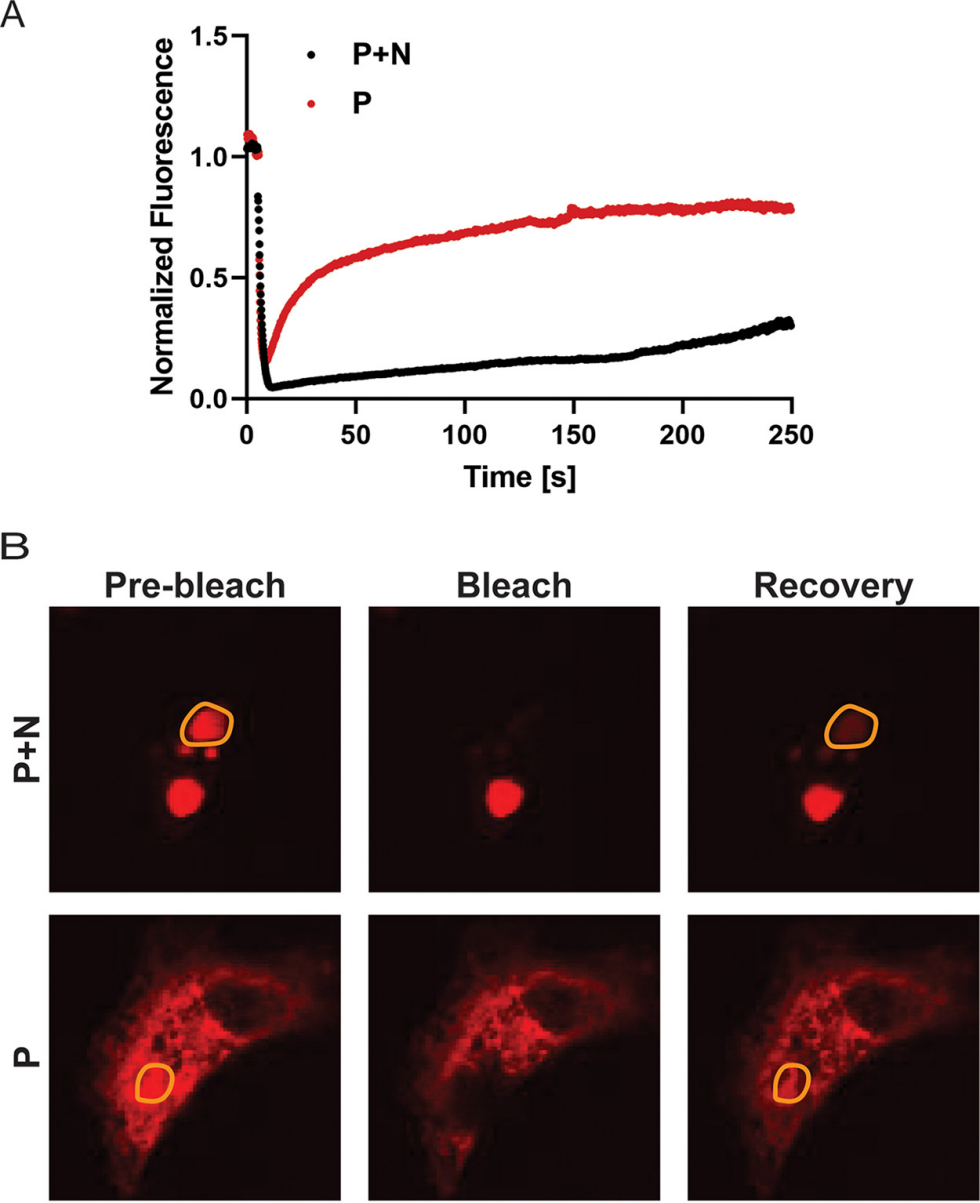
FRAP analysis of HMPV P protein in inclusion bodies and the cytosol. (A) Vero cells were transfected with pCAGGS plasmid expressing mCherry-P only (P) or cotransfected with pCAGGS plasmids encoding mCherry-P and N protein (*P+N*). At 24 h posttransfection, live-cell confocal microscopy was used to perform FRAP at 37°C on punctate regions by drawing a region of interest (ROI) representing a whole inclusion body or an equivalent area in the cytosol with P protein only. FRAP data were corrected for background, were normalized, and are represented as means from the recovery curves. (B) Live-cell confocal images collected during FRAP, showing recovery profiles of inclusions 4 min postbleaching. Bleaching was performed at 100% laser power.

The liquid-like nature of HMPV IB-like structures was analyzed in transfected cells using fluorescence recovery after photobleaching (FRAP) to compare fluorescence recovery rates. When cells were transfected with HMPV P alone, the P protein showed diffuse cytosolic localization and rapid fluorescence recovery (Fig. 2). Alternatively, when cells were transfected with both HMPV N and P to induce IB-like structure formation, HMPV P fluorescence recovery rates in the region of the IB were reduced but recovery was still observed, consistent with what is expected for membraneless liquid-like organelles. This result suggests that interactions between HMPV N and P lead to changes in cellular protein dynamics to form phase-separated regions. Together, these results support the characterization of HMPV IBs as liquid organelles formed by phase separation as sites for efficient replication and transcription.

### Isolated HMPV P phase separates *in vitro*.

**FIG 3:**
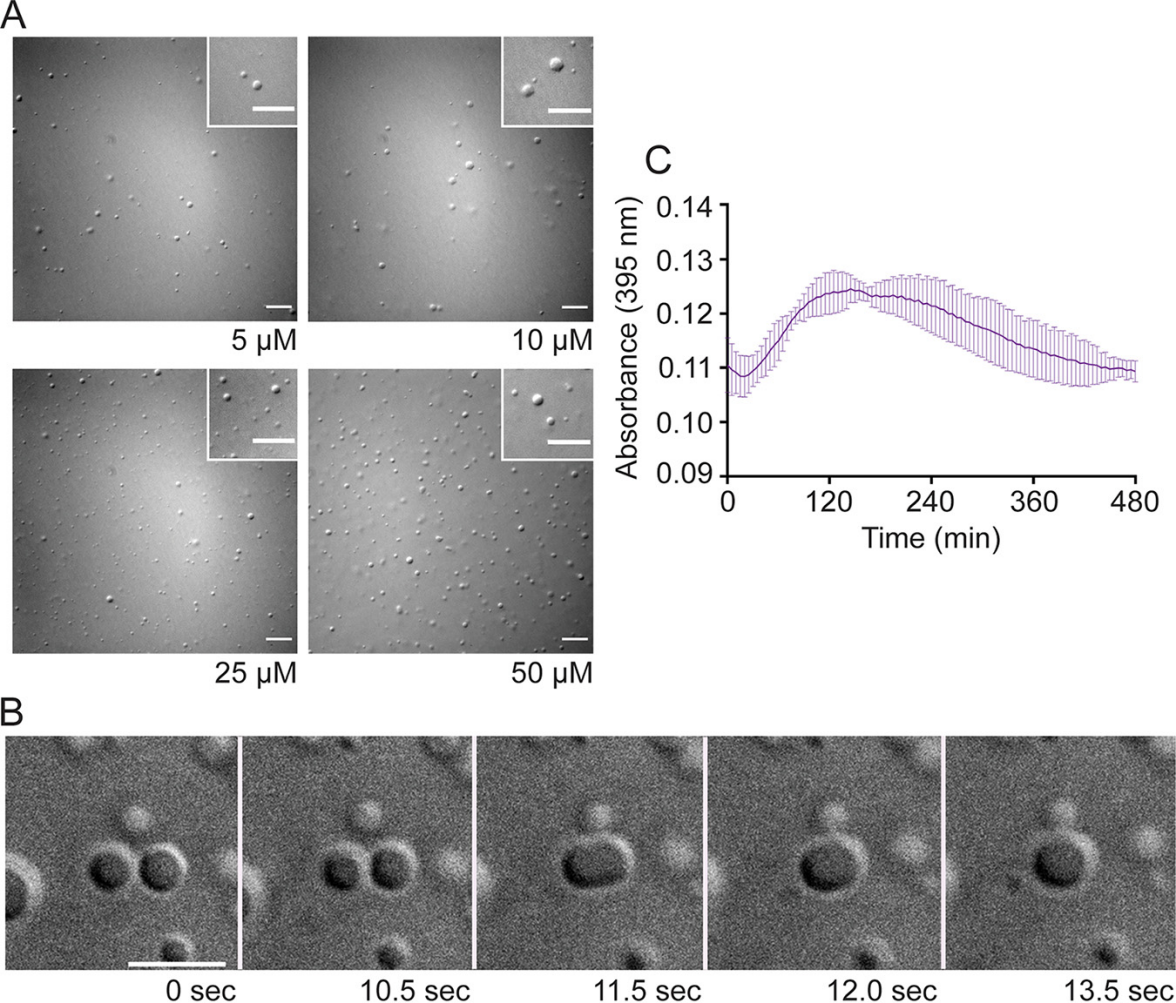
Anion exchange-purified HMPV P phase separates independently *in vitro*. (A) Anion exchange-purified HMPV P was tested at concentrations ranging from 5 μM to 50 μM in a droplet assay (maximum droplet size, 3.4 μm). DIC microscopy imaging of droplets was performed with a 60× objective on a Nikon Eclipse E600 microscope. The scale bar is 10 μm. (B) Time lapse imaging of anion exchange purified HMPV P (80 μM) droplet fusion was acquired using a 100× oil objective on a Zeiss Axiovert 200M microscope. Scale bar, 5 μm. (C) Anion exchange-purified HMPV P (40 μM) was mixed with turbidity assay buffer in a clear 96-well plate. The solution was analyzed using a SpectraMax iD3 instrument to measure the absorbance at 395 nm at 5-min intervals with mixing.

Because HMPV N and P proteins are the minimum requirements for IB-like structure formation in eukaryotic cells, recombinant versions of the proteins were expressed in E. coli and purified for *in vitro* analysis. Purified HMPV P was tested in the presence of the crowding agent dextran, used to mimic cytosolic crowding conditions, to assess its ability to undergo phase separation (24). Phase separation is driven typically by scaffold proteins with specific features that promote multivalent interactions with other proteins or RNA (35–37). HMPV P, which includes long IDRs and alternating charged regions, fits the criteria of a phase separation scaffold protein (27). Unlike the reports for E. coli-expressed MeV and RSV proteins, purified HMPV P formed liquid droplets in the absence of N that were visualized using differential interference contrast (DIC) microscopy, and droplet formation was dependent on the concentration of the P protein (Fig. 3A). Some variation in droplet size was observed at each concentration, potentially due to factors that can affect droplet dynamics, such as local protein or RNA concentration (24).

**FIG 4:**
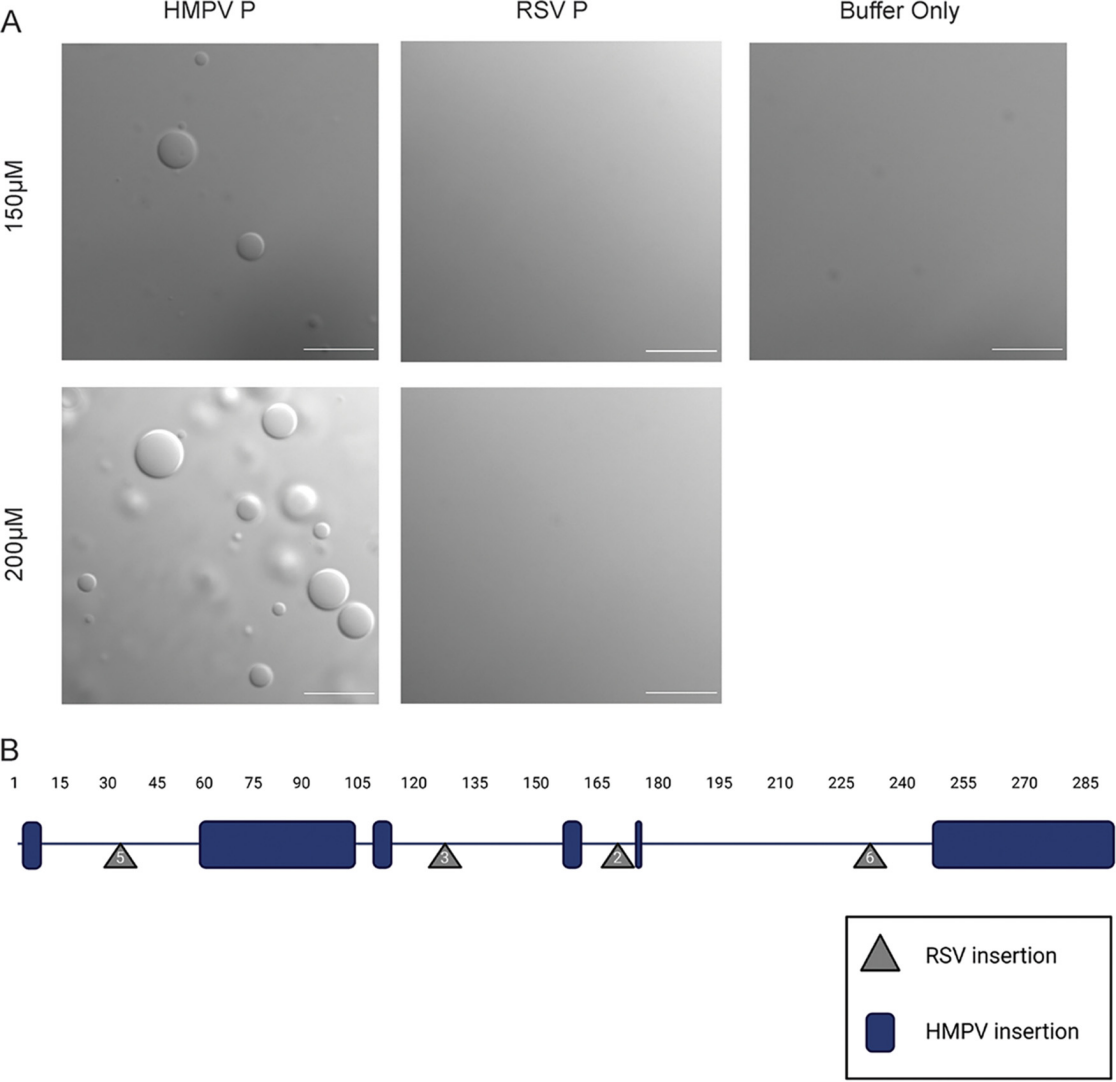
RSV phosphoprotein alone is unable to undergo liquid-liquid phase separation. (A) HMPV P protein was tested parallel with purified RSV A2 P protein, using the same dextran buffer used to mimic the cytosol. RSV P is unable to undergo liquid-liquid phase separation in this study. Droplet assays were performed side by side. DIC microscopy was performed on the Axiovert 200M instrument with a 63× oil objective; scale bar, 10 μm. (B) A schematic of the HMPV P protein amino acid sequence, with representation of residue insertions within HMPV (blue), compared with RSV P, and RSV P insertions (gray), compared with HMPV P. HMPV P (HMPV; Uniprot Q8B9Q8) and RSV (RSV A2; Uniprot P03421) sequences were acquired through the Uniprot database and aligned using the NCBI alignment tool, with adjustments made based on observations in Cardone et al. (45). Made with BioRender.com.

Time lapse imaging of the HMPV P droplets showed that they underwent fusion, consistent with the idea that they possess a liquid nature (Fig. 3B). A turbidity assay was also used to analyze purified HMPV P phase separation. The absorbance of the purified HMPV P protein solution was measured at 395 nm at different time points to detect phase separation. The measurements showed a peak for the absorbance above 0.12 between 2 and 4 h, supporting the microscopy imaging results that HMPV P phase separates in the absence of other viral protein binding partners (Fig. 3C). Dextran buffer alone or purified green fluorescent protein (GFP) in the presence of the dextran-containing buffer was examined (see Fig. S3 in the supplemental material), but no GFP droplet formation was observed, consistent with previous work indicating that GFP-tags can be appended to proteins without affecting phase separation character (38), and that phase separation is not a general characteristic of proteins in the presence of the buffer. Finally, E. coli-expressed and -purified RSV P was tested under the same conditions in a droplet assay side by side with HMPV P. No droplet formation was observed for RSV P when it was tested in isolation (Fig. 4A), consistent with other work on RSV P *in vitro* droplet formation (22), but HMPV P tested at the same time again showed droplet formation, indicating a key difference between HMPV and RSV P.

10.1128/mbio.01099-22.3 FIG S3 Green fluorescent protein in dextran buffer. (A) Bright field and fluorescent images collected on the Axiovert 200M instrument with a 63× oil objective. Droplet formation was not seen under either condition tested. This experiment was performed side by side with HMPV P (200 μM) in the dextran buffer as a positive control, which behaved as seen in previous figures. (B) A PAGE gel using the BioRad precision plus ladder, showing a strong concentration of GFP. Download FIG S3, TIF file, 2.8 MB. Copyright © 2022 Boggs et al. 2022 Boggs et al. https://creativecommons.org/licenses/by/4.0/ This content is distributed under the terms of the Creative Commons Attribution 4.0 International license.

### Interactions with nucleic acid modulate HMPV P phase separation dynamics.

**FIG 5:**
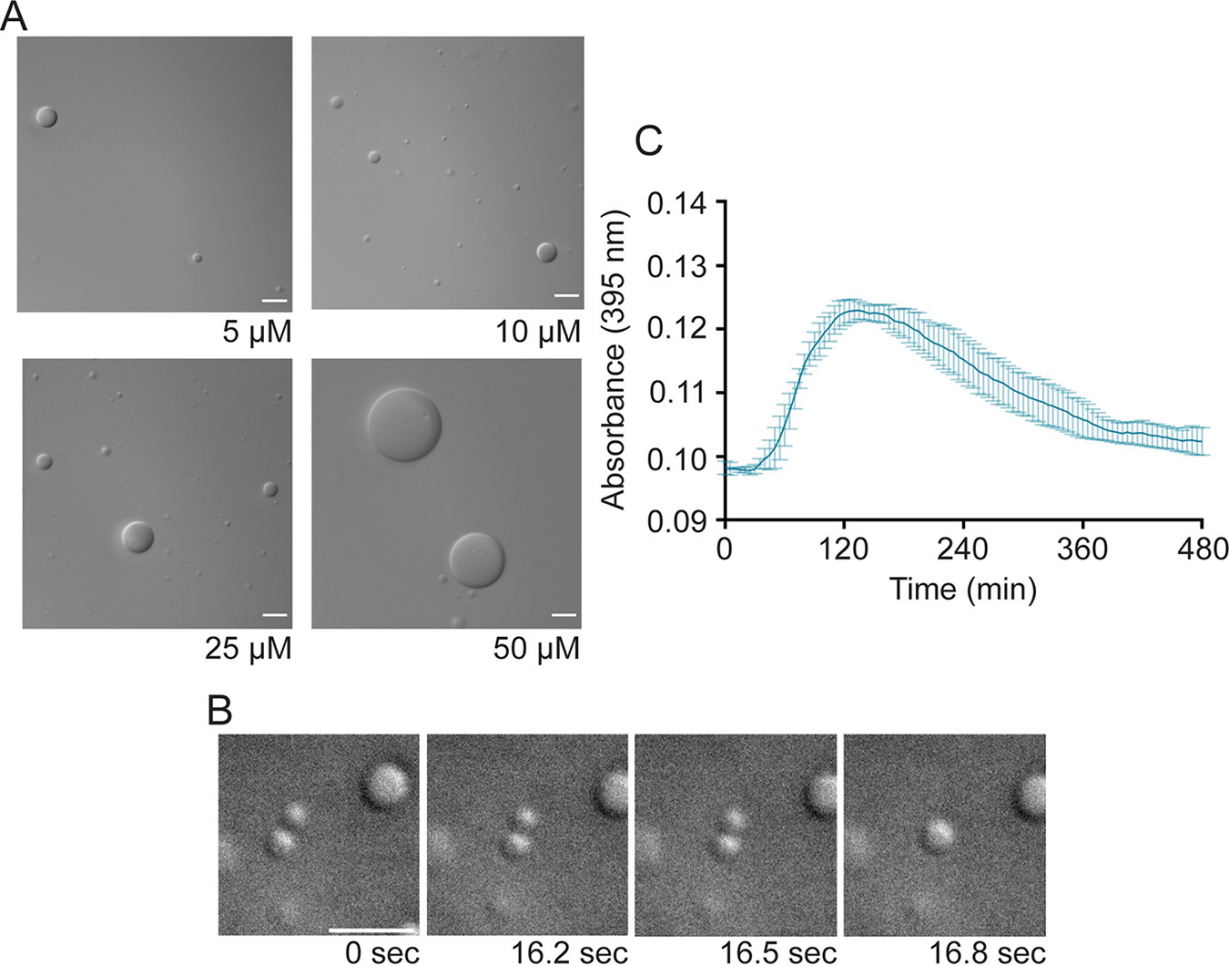
Heparin-purified HMPV P phase separates independently *in vitro*. Heparin-purified HMPV P was tested at concentrations ranging from 5 μM to 50 μM in a droplet assay (maximum droplet size, >50 μm). DIC microscopy imaging of droplets was performed with a 60× objective on a Nikon Eclipse E600 microscope. Scale bar, 10 μm. (B) Time-lapse imaging of heparin-purified HMPV P (150 μM) droplet fusion was acquired using a 100× oil objective on a Zeiss Axiovert 200M microscope. Scale bar, 5 μm. (C) Heparin-purified HMPV P (40 μM) was mixed with turbidity assay buffer in a clear 96-well plate. The solution was analyzed using a SpectraMax iD3 instrument to measure the absorbance at 395 nm at 5-min intervals with mixing.

**FIG 6:**
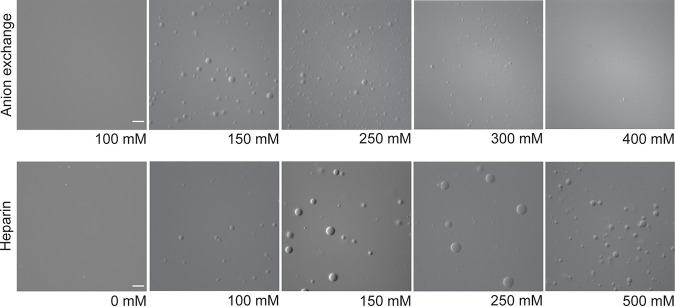
The presence of nucleic acid modulates HMPV P phase-separation dynamics. Anion exchange-purified HMPV P (15 μM) and heparin-purified HMPV P (15 μM) were tested in a droplet assay using buffers with different concentrations of KCl ranging from 0 mM to 500 mM. The DIC microscopy imaging of droplets was performed with a 60× objective on a Nikon Eclipse E600 microscope. Scale bar, 10 μm; the magnification is the same for all images.

Using the protein purification protocol described above, we noticed that the *A*_260/280_ ratio was approximately 1.08, suggesting that the HMPV P protein sample contained nucleic acid. Since nucleic acids are known to play a role in phase separation, we utilized an alternative purification protocol to determine if removing the nucleic acid would influence HMPV P liquid droplet formation. The alternative protocol included treatment with Benzonase nuclease and an immobilized metal affinity chromatography (IMAC) purification step followed by a heparin affinity column purification. This method was successful in removing some of the nucleic acid, as indicated by the decreased *A*_260/280_ ratio of 0.85. Interestingly, the DIC microscopy analysis showed that the recombinant HMPV P protein purified by our alternative protocol formed larger liquid droplets than the original protein sample (Fig. 5A). In addition, time-lapse imaging analysis showed that the liquid droplets were capable of fusing (Fig. 5B). Turbidity assay results for the heparin-purified HMPV P protein were similar to those for previous samples, with a peak above 0.12 between 2 and 4 h (Fig. 5C). These results suggest that the presence of increased levels of nucleic acid modulate HMPV P phase separation dynamics. Charge interactions are known to influence phase separation and nucleic acid binding, so both versions of purified HMPV P (anion exchange purified and heparin purified) were analyzed for liquid droplet formation using buffers with different concentrations of potassium chloride (KCl) ranging from 0 to 500 mM. For the anion exchange-purified HMPV P, liquid droplets were detected easily between 150 and 250 mM KCl. However, droplet formation was inhibited at concentrations below or above that range (Fig. 6). In contrast, the heparin-purified P protein was able to form droplets with as little as 100 mM KCl and as much as 500 mM KCl (Fig. 6). For the heparin-purified HMPV P, the largest droplets formed at a 150 mM KCl droplet size ranging from 0.5 to 5 μm, with an average of 0.64 μm. Average IB size was lower, comparatively, in 250 mM salt (average droplet size of 2.5 μm), and similar results were observed at the 500 mM concentration. The anion-exchanged protein also formed the largest droplets at the 150 mM concentration with an average size of 2.36 μm ranging from 0.57 μm to 6.8 μm. The anion-purified P protein also formed smaller droplets with increasing salt concentration above 150 mM, with an average diameter of 1.7 μm at 250 mM KCl and 1 μm at 300 mM KCl. Droplets were consistently smaller for the anion exchange-purified protein in contrast to the heparin-purified product at comparable salt concentrations. These results suggest that HMPV P protein samples containing higher levels of nucleic acid are more sensitive to changes in charge, thus leading to the disruption of liquid droplet formation *in vitro*.

### HMPV P recruits N^0^-P to liquid droplets.

**FIG 7:**
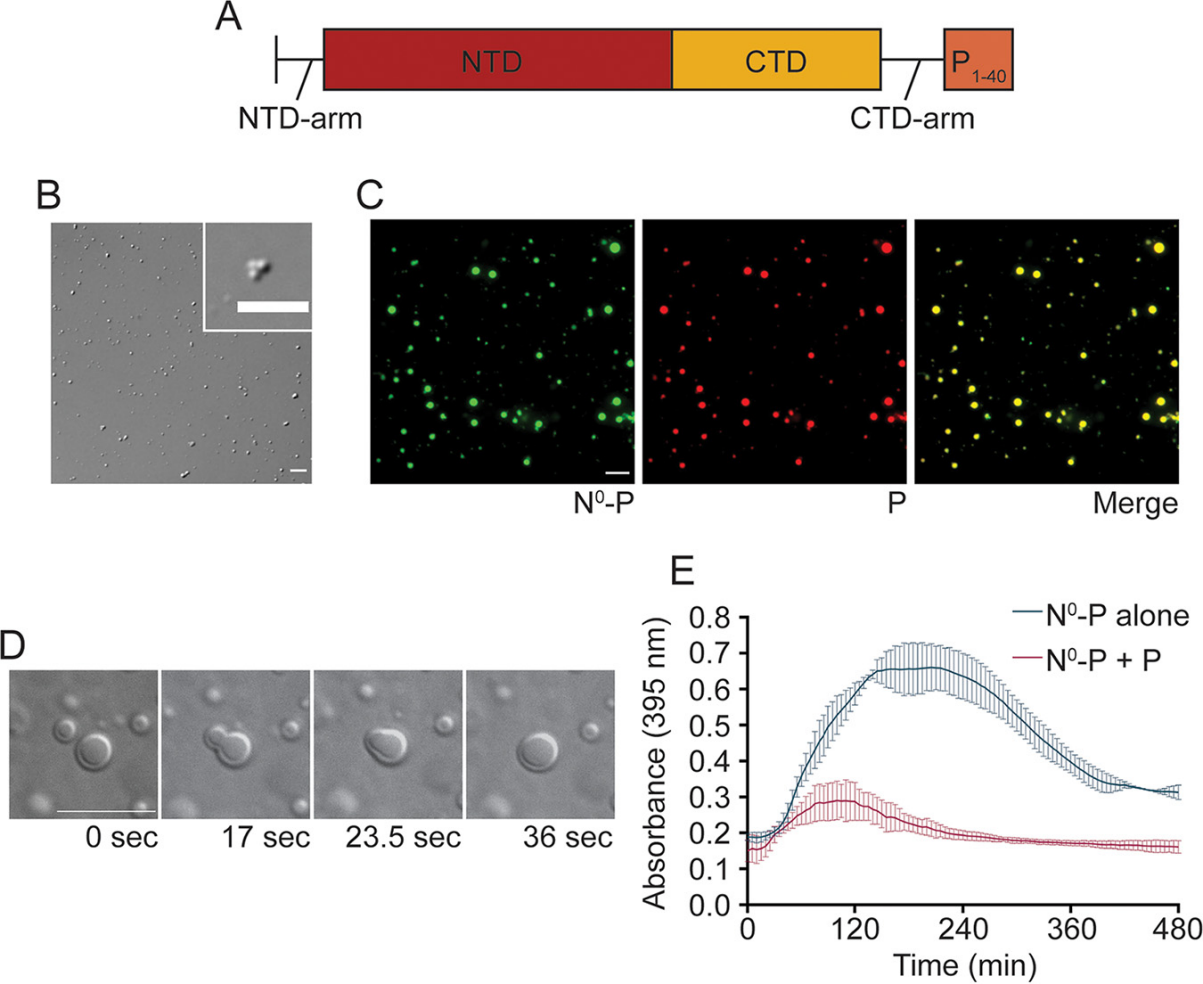
HMPV P recruits N^0^-P to liquid droplets. (A) Schematic of the N^0^-P construct which includes full-length HMPV N fused to the first 40 amino acids of HMPV P. (B) HMPV N^0^-P (15 μM) was tested in a droplet assay. DIC images were acquired at different time points using a 60× objective on a Nikon Eclipse E600 instrument. Scale bar, 7 μm. (C) HMPV N^0^-P (15 μM) labeled with Alexa 488 TFP ester was mixed with anion exchange-purified HMPV P (15 μM) labeled with Alexa 594 NHS ester in a droplet assay. Fluorescence images were acquired using a 60× objective on a Nikon Eclipse E600 instrument. Scale bar, 10 μm. (D) HMPV N^0^-P (50 μM) was mixed with anion exchange-purified HMPV P (50 μM). Time-lapse imaging of N^0^-P/P droplet fusion was acquired using a 100× oil objective on a Zeiss Axiovert 200M microscope. Scale bar, 10 μm. (E) HMPV N^0^-P (40 μM) was tested alone or with anion exchange-purified HMPV P (40 μM) in a turbidity assay. The protein solutions were plated in a clear 96-well plate with turbidity assay buffer, and the absorbance was measured at 395 nm by a SpectraMax iD3 instrument at 5-min intervals.

WT HMPV N spontaneously oligomerizes and binds to nonspecific RNAs during standard purification procedures (29). Thus, we utilized a recombinant N^0^-P construct that includes full-length N (1 to 394) fused to a P peptide (1 to 40) to maintain N in a monomeric, RNA-free form for purified protein analysis (Fig. 7A), a strategy that had been successfully utilized by Renner et al. (29). The N^0^-P construct purified by IMAC formed gel-like structures that clumped together in irregular shapes that were visualized by DIC microscopy (Fig. 7B). Unlike anion exchange-purified HMPV P, the gel-like HMPV N^0^-P structures remained partially undissolved in 500 mM KCl (data not shown). Over time, these gel-like structures aggregated together but did not undergo fusion (Fig. 7B). In agreement with our microscopy results, turbidity assays performed with the IMAC-purified N^0^-P protein gave high absorbance readings that peaked above 0.6, further indicating that the gel-like structures were aggregating in solution (Fig. 7E). The subsequent drop in absorbance suggests that the aggregates settled to the bottom of the 96-well plate.

The N^0^-P construct was examined in combination with anion exchange-purified HMPV P using a droplet assay to determine if the P protein could influence N^0^-P dynamics in solution. DIC and fluorescence microscopy analyses showed that mixing the two proteins led to enhanced phase separation as indicated by the presence of larger and more numerous droplets than we observed previously for HMPV P alone. N^0^-P and P were incorporated into the same liquid droplets, as indicated by the colocalization of the fluorescent signals used to label the proteins (Fig. 7C). In addition, the phase-separated droplets underwent fusion events (Fig. 7D). A turbidity assay was utilized to determine if combining N^0^-P with P affected the absorbance of the solution. The results showed that compared with N^0^-P alone, the combination of N^0^-P and P led to lower absorbance readings that peaked around 0.3 at 2 h (Fig. 7E). Together, these findings support that HMPV P facilitates interactions with N^0^-P to recruit the protein into liquid droplets and interactions between the proteins prevent the N^0^-P construct from transitioning to a gel-like state.

### HMPV P recruits N-RNA rings to liquid droplets.

**FIG 8:**
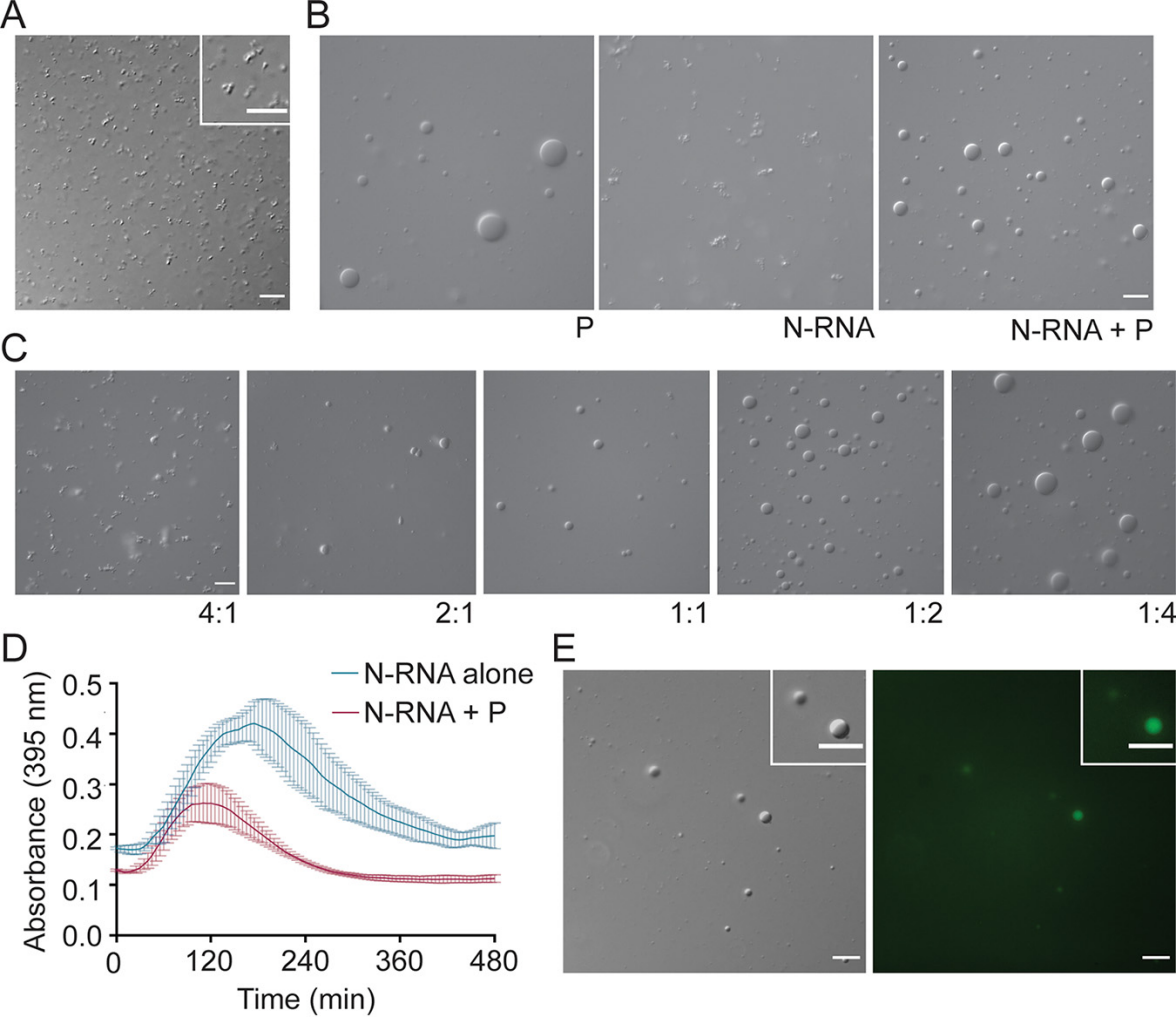
HMPV P recruits N-RNA rings to liquid droplets. (A) HMPV N-RNA (25 μM) was tested in a droplet assay. The DIC microscopy imaging of droplets was performed with a 60× objective on a Nikon Eclipse E600 microscope. Scale bar, 10 μm. (B) HMPV N-RNA (15 μM) was mixed with heparin-purified HMPV P (15 μM) in a droplet assay. DIC images were acquired using a 60× objective on a Nikon Eclipse E600 instrument. Scale bar, 10 μm. (C) HMPV N-RNA and heparin-purified HMPV P were tested in a droplet assay at different ratios (4:1 for 20 μM N-RNA: 5 μM P; 2:1 for 10 μM N-RNA: 5 μM P; 1:1 for 5 μM N-RNA: 5 μM P; 1:2 for 5 μM N-RNA: 10 μM P; 1:4 for 5 μM N-RNA: 20 μM P). The DIC microscopy imaging of droplets was performed as described above. (D) HMPV N-RNA (40 μM) was tested alone or with heparin-purified HMPV P (40 μM) in a turbidity assay. The protein solutions were plated in a clear 96-well plate with turbidity assay buffer, and the absorbance was measured at 395 nm by a SpectraMax iD3 instrument at 5-min intervals with mixing. (E) HMPV N-RNA (15 μM), heparin-purified HMPV P (15 μM) and an RNA decamer tagged with 6-carboxyfluorescein on the 3′ end (5 μM) were mixed and tested in a droplet assay. The DIC and fluorescence microscopy imaging of droplets were performed as described above. Scale bar, 10 μm.

In addition to the monomeric N^0^-P construct, WT N-RNA rings were purified for phase separation analysis in the presence or absence of HMPV P. DIC imaging of N-RNA rings in the droplet assay showed the formation of clumped, irregularly shaped structures that did not undergo fusion, suggesting that this protein-RNA complex does not form liquid-like phase-separated structures independently (Fig. 8A). Combining the purified N-RNA rings with heparin-purified HMPV P resulted in N-RNA complex incorporation into liquid droplets (Fig. 8B). The N-RNA/P droplets (maximum droplet size, 6 μm) were generally smaller than the P alone droplets (maximum droplet size, 11 μm), suggesting that this combination influences phase separation dynamics. The influence of HMPV P on N-RNA for liquid droplet formation was reflected in the turbidity assay results which showed a lower peak for absorbance around 0.2 at 2 h than the absorbance for N-RNA alone (Fig. 8D).

HMPV P and N-RNA were tested in our *in vitro* system at different ratios to determine the conditions that were required for N-RNA to be recruited to liquid droplets. Although N-RNA aggregates were still present at 4:1 and 2:1 ratios of N-RNA/P, round droplets were detected easily at a 1:1 ratio. The number and size of the round droplets increased in samples with a higher proportion of HMPV P (1:2 and 1:4) (Fig. 8C). These results suggest that a specific ratio of N-RNA/P must be met before N-RNA is induced to phase separate into droplets. Liquid droplets containing HMPV P and N-RNA were also shown to incorporate a fluorescent RNA oligomer (Fig. 7E). These findings highlight that HMPV P, N, and RNA form complex multivalent interactions to promote phase separation and to support the structure of IBs required to enhance replication and transcription.

### HMPV P recruits RNA-binding mutant N K171A/R186A to gel-like droplets.

**FIG 9:**
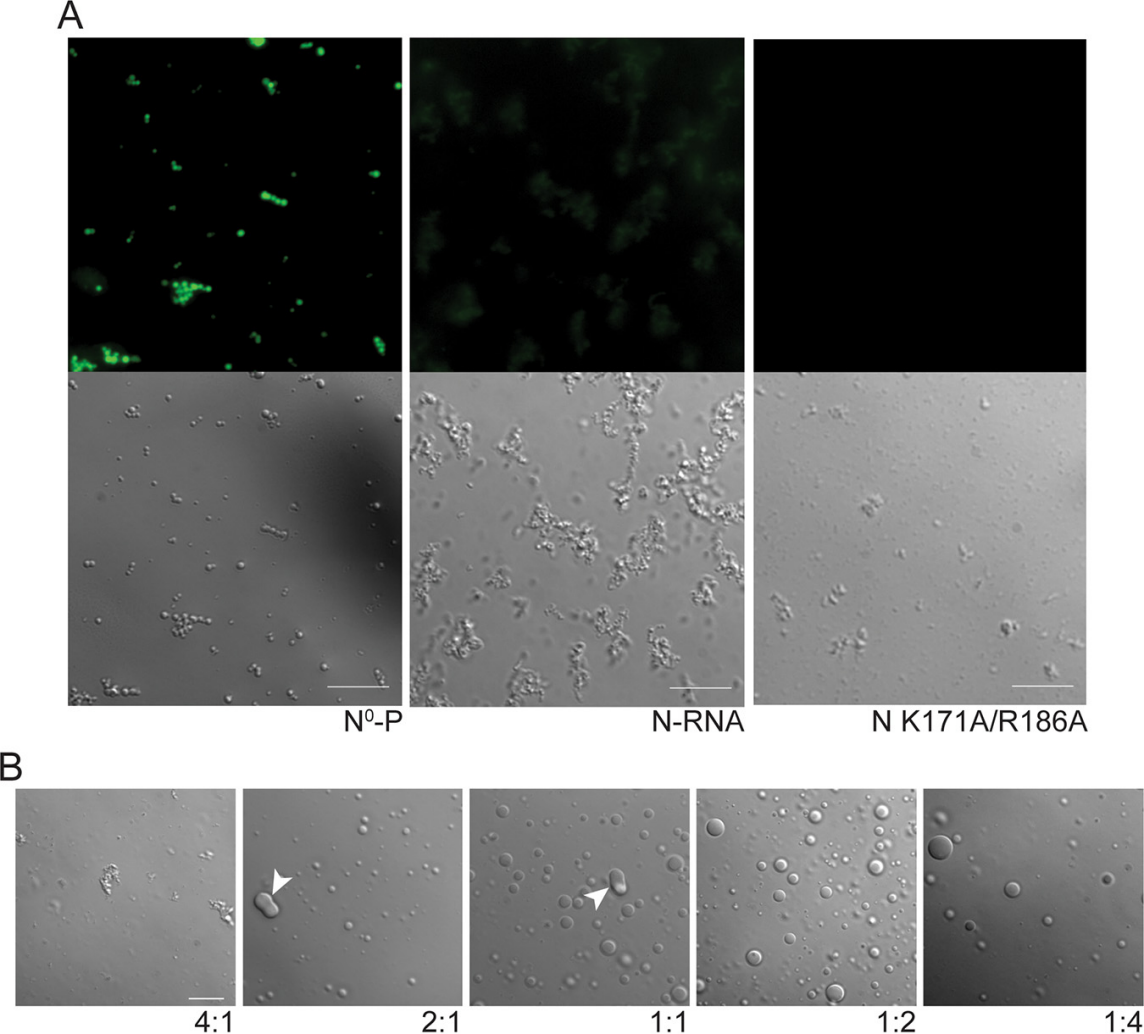
RNA-binding mutant HMPV N K171A/R186A forms gel-like droplets with P. (A) HMPV N^0^-P, N-RNA, or N K171A/R186A (15 μM) were tested in a droplet assay with an RNA decamer tagged with 6-carboxyfluorescein on the 3′ end (5 μM). DIC and fluorescence microscopy imaging were performed on a Zeiss Axiovert 200M instrument with a 63× oil objective. Scale bar, 10 μm. (B) HMPV N K171A/R186A and heparin-purified HMPV P were tested in a droplet assay at different ratios (4:1 for 20 μM N K171A/R186A: 5 μM P; 2:1 for 10 μM N K171A/R186A: 5 μM P; 1:1 for 5 μM N K171A/R186A: 5 μM P; 1:2 for 5 μM N K171A/R186A: 10 μM P; 1:4 for 5 μM N K171S/R186A: 20 μM P). The DIC microscopy imaging of droplets was performed on a Zeiss Axiovert 200M microscope with a 63× oil objective. White arrowheads indicate altered droplet fusion. Scale bar, 10 μm.

An HMPV N mutant (K171A/R186A) was generated to determine if RNA binding affects N recruitment into droplets. This mutant was designed based on the RSV construct N K170A/R185A which lacks the ability to bind RNA (39). HMPV N^0^-P, N-RNA, and N K171A/R186A were tested individually with a fluorescent RNA oligomer in a droplet assay to assess the RNA-binding capabilities of the different constructs. The RNA oligomer incorporated into N^0^-P structures, suggesting that the RNA may disrupt binding of the P_1-40_ peptide to N (Fig. 9A). Alternatively, the P_1-40_ peptide, which contains positively charged residues, may interact directly with the RNA oligomer. In contrast to N^0^-P, N-RNA showed a weak interaction with the fluorescent RNA oligomer, suggesting that the oligomer poorly disrupts the existing interactions between HMPV N and RNA in stable ring structures (Fig. 9A). The N K171A/R186A mutant showed no colocalization with the fluorescent RNA oligomer, suggesting that the mutation of these residues in the RNA-binding cleft of HMPV N effectively inhibits RNA binding or significantly slows the exchange between any bound RNA and the fluorescent RNA oligomer (Fig. 9A).

Subsequently, HMPV N K171A/R186A was tested at different ratios with heparin-purified HMPV P in a droplet assay to analyze phase-separation dynamics (Fig. 9B). At a 4:1 ratio of N K171A/R186A:P, microscopy imaging showed the presence of aggregates and no liquid droplets. Droplets became visible as the concentration of HMPV P was increased relative to the N RNA-binding mutant. However, the droplets exhibited gel-like features, and droplets were seen to adhere to each other (Fig. 8A) but were unable to undergo fusion. This finding suggests that RNA binding to HMPV N or the presence of charged surfaces due to the presence of RNA plays an important role in modulating phase-separation dynamics *in vitro* and supports our model that HMPV P, N, and RNA form complex interactions in cells to promote replication and transcription.

## DISCUSSION

IB formation has been reported for many negative-strand viruses across the *Mononegavirales* order (40). Recent evidence supports that these structures function as viral factories by concentrating the materials required for replication and transcription. Cellular studies of IBs have shown that these structures are membraneless and dynamic, which led to the characterization of IBs as liquid organelles. Although membraneless organelles have been recognized in the cell for decades, the formation of these structures has been linked only recently to the process of phase separation. Understanding the role of phase separation in IB formation may be critical for discovering new targets for pan-antiviral development. Until now, no reports have been published to determine if HMPV IBs are consistent with phase-separated liquid organelles. In this study, cellular experiments were utilized to analyze the dynamic nature of HMPV IBs in infected or transfected cells. Additionally, *in vitro* experiments were performed to test recombinant versions of HMPV, E. coli-expressed, purified proteins in phase-separation assays. These studies provide strong evidence for the novel role of HMPV P as a scaffold for recruiting N protein, and other components, to phase-separated regions.

Cellular analysis of HMPV IBs, using live-cell imaging and FRAP, showed that these structures form as distinct phase-separated regions that exchange components with the surrounding cytoplasm and undergo fusion and fission. Interestingly, our analysis of cells infected with recombinant HMPV-mCherryP virus showed that levels of IB fusion remained stable from 24 to 72 hpi, whereas fission events increased significantly during this time frame (Fig. 1). Since IB diameter increased significantly from 24 to 72 hpi, this finding suggests that early fusion events and the incorporation of cellular components, newly synthesized HMPV proteins, and viral RNA contribute to the growth of IBs over time. Furthermore, the plateau of fusion events suggests that IBs likely mature to a gel-like state by 72 hpi. In contrast, fission levels may be less affected by the gel-like state due to the activity of vesicles that traffic IB components out of large IBs, as we have observed via live-cell imaging (data not shown). IB fission events may be linked to the release of small replication bodies to create new viral factories (15). Alternatively, fission may promote the formation of unique IB subpopulations later in infection to facilitate assembly and budding (41). These findings highlight the crucial role IBs likely play in establishing and promoting the spread of infection.

To analyze the protein interactions that govern IB formation, we compared purified HMPV P in the presence or absence of nucleic acid with a set of N forms using *in vitro* phase-separation experiments. Importantly, we found that HMPV P phase separates independently *in vitro* and can act as a scaffold to recruit other client proteins, including HMPV N, to liquid droplets to drive phase separation. Additionally, our findings suggest a previously undescribed role for HMPV P in interacting with RNA, an interaction that modulates phase separation. As the genome and antigenome of HMPV are fully coated by N, P could interact with viral mRNAs or with cellular RNAs in the context of an infection. Features of viral P proteins, including long regions of intrinsic disorder, match the molecular signature of proteins that phase separate under physiological conditions (23, 27). Furthermore, they are consistent with HMPV P as a scaffold protein that binds a variety of substrates, such as viral proteins and RNA, to promote IB formation. The recently published structure of the HMPV polymerase/P protein complex highlights the importance of IDRs in allowing HMPV P to adopt a variety of binding conformations (31, 42). The propensity for HMPV P to mediate multivalent interactions and phase separate independently *in vitro* suggests that it regulates similar functions for IB formation during infection (35).

In contrast to our *in vitro* results, the HMPV N and P proteins must be coexpressed in cells to generate IB-like structures (25). Without HMPV N, the P protein showed both diffuse cytoplasmic localization and peripheral filopodia-like localization (25). This difference between the cellular and *in vitro* systems suggests that host factors in the cytoplasm may block HMPV P interactions required to induce phase separation. Coexpression of HMPV P and N likely acts to initiate phase separation in cells by concentrating enough IB components to drive phase separation. Phase separation is a highly sensitive process that depends on factors such as protein/RNA concentration, salt content, post-translational modifications, pH, and temperature (23). One or more of these factors may prevent HMPV P from phase separating when expressed independently in cells. Furthermore, results showed that removal of nucleic acid from purified HMPV P modulated liquid droplet formation *in vitro* (Fig. 5), suggesting that HMPV P phase separation in cells may be impacted significantly by the presence of RNA. Additionally, during HMPV infection, the N protein is always expressed in excess compared with HMPV P due to the location of the N gene within the viral genome. This result suggests that HMPV P may lack opportunities for independent phase separation during infection due the local concentration of other viral factors involved in IB formation. Although *in vitro* studies are crucial for deciphering the mechanisms of HMPV phase separation, the increased complexity of protein and RNA interactions during cellular infection must always be considered.

Our work is the first to provide evidence of a viral P protein functioning as a phase-separation scaffold, in contrast to related systems. Recent reports on MeV and RSV showed that a combination of the E. coli-expressed N and P proteins were required to induce droplet formation *in vitro* (20, 21), and our work also shows the absence of phase separation when RSV P is examined in isolation (Fig. 4A). Although *Mononegavirales* P proteins share common structural features, they lack sequence similarity and vary in length (43). MeV P is 213 residues longer than HMPV P and includes a folded C-terminal (XD) domain after the unfolded P_loop_. The pneumoviral RSV and HMPV P proteins possess a similar domain organization, but sequence differences likely promote unique phase-separation interactions for each virus. For instance, HMPV P is 53 residues longer than RSV P and contains insertions in the N-terminal and C-terminal domains that may influence IDR behavior (43) (Fig. 4B; see Fig. S4 in the supplemental material). The C-terminal domain and oligomerization domain of RSV P were required for liquid droplet formation with N-RNA, suggesting that the acidic insertion in the HMPV P C-terminal domain may modulate phase separation (21, 43). The differences observed for these viral systems emphasize that phase separation is highly dependent on multivalent interactions mediated by the unique composition of the P protein.

10.1128/mbio.01099-22.4 FIG S4 RSV A2 phosphoprotein residue alignment with HMPV CAN98-73 phosphoprotein. HMPV P (HMPV; Q8B9Q8) and RSV (RSV A2; P03421) sequences were acquired through the Uniprot database and aligned using the NCBI alignment tool, with alignment adjustments based on discoveries by Cardone et al. (45). Made with BioRender.com. Download FIG S4, TIF file, 1.1 MB. Copyright © 2022 Boggs et al. 2022 Boggs et al. https://creativecommons.org/licenses/by/4.0/ This content is distributed under the terms of the Creative Commons Attribution 4.0 International license.

The purified P proteins studied in our work and in work on RSV P (21) and measles virus P (20) were expressed in E. coli and thus may lack post-translational modifications, such as phosphorylation. The viral P proteins, as so named due to the presence of phosphorylation at multiple locations on the protein, and previous studies on RSV (44–46) and *Sf9-*expressed HMPV P (31) indicate that pneumovirus P proteins are phosphorylated. However, no information about the sites of phosphorylation utilized during HMPV infection is available, nor is it known if these sites change over the course of infection. Measles virus inclusion body size was shown to be influenced by P protein phosphorylation and the host enzyme casein kinase 2 (19), suggesting a potential link between post-translational modifications of P and the dynamics of inclusion body formation. In addition, a number of studies in multiple systems have found a role for phosphorylation in the control of phase separation (19, 35, 47, 48). Thus, future experiments to determine how P protein phosphorylation modulates over the course of infection and how these post-translational modifications impact related phase separation represent an exciting direction, and they could be paired with studies of purified proteins containing the same sets of phosphorylation sites to assess the impact of each utilized site on phase separation dynamics.

Although HMPV N and P are required for IB formation in cells, the role of different N protein forms in phase separation required further exploration. We compared monomeric N protein (N^0^-P) and N-RNA with N K171A/R186A to analyze the effects of oligomerization and RNA binding on phase separation with the HMPV P scaffold. Although all of the N forms were recruited to droplets by HMPV P, the incorporation of N K171A/R186A led to the formation of droplets that failed to undergo complete fusion, suggesting that they were gel-like rather than liquid-like in nature. This finding highlights that RNA interactions with HMPV N and P alter phase-separation dynamics and suggests that viral RNA levels may modulate IB maturation during the course of HMPV infection. A minimal MeV phase-separation *in vitro* system showed that RNA diffuses into MeV N/P liquid droplets, triggering the formation of nucleocapsid-like particles (20). Interestingly, coexpression of a MeV N RNA-binding mutant with P did not alter the morphology of IB-like structures in cells compared to coexpression of WT N and P (19). These findings suggest that RNA binding is not required for MeV IB formation, but RNA incorporation likely serves to enhance ribonucleoprotein assembly during infection. In contrast, a monomeric RNA-free RSV N mutant failed to form IB-like structures with RSV P in cells, suggesting that RSV N must oligomerize and/or bind RNA to mediate IB formation (21). Our *in vitro* results with HMPV N^0^-P and N K171A/R186A confirmed that N protein oligomerization and RNA interactions are not required for phase separation with HMPV P.

Here, we showed that HMPV IBs are liquid organelles and that HMPV P acts as a scaffold to recruit different forms of N to liquid droplets. We report that nucleic acid interactions with P and N alter phase-separation dynamics, suggesting that viral RNA binding plays a significant role in HMPV IB formation and maturation. Recent work on RSV utilized a condensate-hardening drug to block RSV replication in the lungs of infected mice (49). This exciting evidence suggests that IBs of various negative-strand viruses may serve as druggable targets for inhibiting infection. The work presented here builds on the foundation for understanding the formation of IBs and the mechanisms that regulate phase separation for negative-strand viruses. Deciphering the protein and RNA interactions that influence IB phase separation will be essential for the development of pan-antiviral drugs to target viral factories.

## MATERIALS AND METHODS

### Construction of a recombinant HMPV-mCherryP virus.

The plasmids encoding the full-length genome sequence of HMPV strain JPS02-76 (p+JPS07E2) and the accessory proteins N, M2-1, L, and P (pCITE-76N, -76M2-1, -76L, and -76P) were kindly provided by Makoto Takeda (National Institute of Infectious Diseases, Tokyo, Japan) (34). To insert the mCherryP cassette within the p+JPS07E2 plasmid, a vector containing the partial sequence of N followed by the N terminus mCherry-tagged P sequence, flanked by NheI and SacI restriction sites, was synthesized (GenScript). The sequence within this vector was then subcloned into p+JPS07E2 using the NheI and SacI restriction sites. The correct insertion of the cassette into the plasmid was verified by sequencing. To rescue the recombinant HMPV, the methodology described by Shirogane et al. (34) was used. Briefly, BSR cells stably expressing the T7 RNA polymerase were transfected with plasmids p+JPS07E2 and pCITE-76N, -76M2-1, -76L, and -76P using Lipofectamine3000, following manufacturer instructions. Forty-eight hours posttransfection, the cells were scraped from the plate onto the medium, and half of the volume was overlaid onto a monolayer of Vero cells, in Opti-MEM with 0.3 μg/mL tosylsulfonyl phenylalanyl chloromethyl ketone-trypsin (TPCK-Try). The medium was replaced every other day until extensive cytopathic effect and fluorescent signal were observed. Cells and media were then recovered and used to propagate the passage 1 of the recombinant virus in Vero cells, as described previously.

### Fluorescence recovery after photobleaching (FRAP).

Vero cells were transfected with a pCAGGS plasmid expressing mCherry-P only (P) or cotransfected with pCAGGS plasmids encoding mCherry-P and N protein (*P+N*). Twenty-four hours post-transfection, live-cell confocal microscopy was used to perform FRAP at 37°C on punctate regions by drawing a region of interest (ROI) representing a whole inclusion body or an equivalent area in the cytosol with the P protein only. Imaging was completed on the Nikon A1R confocal microscope, using a Plan Fluor 40× oil DIC objective. For photobleaching, a laser wavelength of 405 nm with a laser power setting of 100% was utilized. Each experiment used 5 s of prebleaching acquisition, with 4 to 5 min of recovery.

### Expression and purification of HMPV P.

The CAN97-83 HMPV P construct was cloned into the plasmid pET 302/NT-His between the cleavage sites EcoRI and XhoI and expressed in BL21(DE3) CodonPlus RIL cells (Agilent) overnight at 37°C in terrific broth (TB) containing ampicillin after induction at an optical density (OD) of 1.4 with 1 mM isopropyl-β-d-thiogalactopyranoside (IPTG). Cells were lysed with 20 mM Tris and 200 mM NaCl (pH 7.5) containing cOmplete EDTA-free protease inhibitor cocktail (Sigma) and 125 μg/mL lysozyme. After cells were incubated on ice for 20 min, the solution was sonicated three times at 60% intensity for 15 sec. The lysate was spun at 18,000 rpm for 30 min at 4°C. The crude lysate rocked with His-select nickel affinity gel resin (Sigma) for 45 min at 4°C. The resin was washed one time with lysis buffer and two times with 20 mM Tris, 200 mM NaCl, and 20 mM imidazole (pH 7.5). The protein was eluted with 20 mM Tris, 200 mM NaCl, and 250 mM imidazole (pH 7.5). The eluate was loaded onto a HiTrap Q high-performance (HP) anion-exchange chromatography column (Cytiva). The column was washed with 20 mM Tris (pH 7.5). Then, fractions were eluted with 20 mM Tris and 1 M NaCl (pH 7.5). The fractions containing HMPV P were concentrated, and buffer was exchanged into 25 mM HEPES and 150 mM KCl (pH 7.5) using a PD-10 desalting column with Sephadex G-25 resin (GE Healthcare).

To reduce nucleic acid binding, some HMPV P lysates were treated with Benzonase during the cell lysis step. Instead of anion exchange, the His-select purification was followed by heparin purification using a HiTrap heparin HP column with an increasing NaCl gradient from 200 mM to 1 M prior to buffer exchange with the PD-10 column. After buffer exchange, the protein was concentrated, flash frozen, and stored at −80°C. Protein purity was then examined via PAGE (see Fig. S2 in the supplemental material).

10.1128/mbio.01099-22.2 FIG S2 Respiratory syncytial virus A2 P protein PAGE. MBP-6×-His-RSV A2 phosphoprotein (317 μM) expressed in BL21 (DE3) E. coli; immobilized on nickel resin; and further purified using amylose, ion exchange Q column, and a gel filtration column. The RSV P MBP cut site was digested by TEV, leaving the RSV A2 P protein. The HMPV phosphoprotein (979 μM) was expressed and purified as in Fig. S1 in the supplemental material. Bands below protein are degraded HMPV P, as verified through mass spectrometry. Download FIG S2, TIF file, 2.6 MB. Copyright © 2022 Boggs et al. 2022 Boggs et al. https://creativecommons.org/licenses/by/4.0/ This content is distributed under the terms of the Creative Commons Attribution 4.0 International license.

10.1128/mbio.01099-22.1 FIG S1 Purified protein PAGE. (A) HMPV 6×-His-phosphoprotein (979μM) expressed in BL21 (DE3) E. coli, immobilized on nickel resin, and further purified using a heparin column. The final protein *A*_260/A280_ was 1.15. Other bands found in the final product were analyzed using mass spectrometry, and all were shown to be HMPV phosphoprotein portions. (B) HMPV 6×-His-nucleoprotein (137μM) was expressed and purified as in A. The final protein *A*_260/A280_ was 0. 8. (C) HMPV 6×-His-nucleoprotein K171A/R186A (66 μM) was expressed and purified as in A. The final protein *A*_260/280_ was 0.96. (D) HMPV 6×-His-N^0^-P (169μM) was expressed and purified as in A. The final protein *A*_260/280_ was 1.08. (E) HMPV 6×-His-phosphoprotein (169 μM) was expressed as in A, immobilized on nickel resin, and further purified using anion exchange. The final protein *A*_260/280_ was 1.08. An *A*_260/280_ ratio of 0.6 is generally considered pure for protein. Download FIG S1, TIF file, 2.5 MB. Copyright © 2022 Boggs et al. 2022 Boggs et al. https://creativecommons.org/licenses/by/4.0/ This content is distributed under the terms of the Creative Commons Attribution 4.0 International license.

### Expression and purification of HMPV N^0^-P.

The CAN97-83 HMPV N^0^-P construct with a 6× C-terminal His_6_ tag was synthesized by GenScript in the pET-29b(+) plasmid and cloned between the NdeI and KpnI cleavage sites. The construct was expressed in E. coli Rosetta 2(DE3) competent cells (Novagen) overnight at 18°C in TB containing kanamycin after induction at an OD of 0.8 with IPTG. Cells were lysed (20 mM Tris, 500 mM NaCl, 10 mM imidazole [pH 7], protease inhibitor, lysozyme, and 250 units of benzonase [Sigma]) and incubated on ice for 20 min. The solution was sonicated and spun as described above except the lysate spun for 45 min. The crude lysate was incubated with resin as described above. The resin was washed once with 20 mM Tris, 500 mM NaCl, and 10 mM imidazole [pH 7] and twice with 20 mM Tris and 500 mM NaCl [pH 7]; and the protein was eluted with 20 mM Tris, 500 mM NaCl, and 300 mM imidazole (pH 7). The eluate was concentrated and buffer exchanged into 25 mM HEPES and 150 mM KCl (pH 7.5). The protein was concentrated and stored as described above. Protein purity was determined via PAGE (Fig. S2).

### Expression and purification of HMPV N-RNA.

The CAN97-83 HMPV N construct with a 6× C-terminal His_6_ tag was synthesized and cloned into pET-29b(+) as described above. The construct was expressed and induced as described for N^0^-P. Cells were lysed (25 mM Tris, 1 M NaCl [pH 8], protease inhibitor, lysozyme, and Benzonase) and treated as described above except the lysate spun for 1 h. The crude lysate was loaded onto a column containing preequilibrated resin at 4°C, washed two times (25 mM Tris and 1 M NaCl [pH 8]), and eluted (25 mM Tris, 1 M NaCl, and 400 mM imidazole [pH 8]). The eluate was concentrated, and the NaCl concentration of the sample was adjusted to 100 mM using 25 mM Tris (pH 8). Then, the sample was loaded onto a HiTrap heparin HP column (Sigma) using an increasing NaCl gradient from 200 mM to 1 M. Fractions containing the HMPV N protein were buffer exchanged and stored as described above. Protein quality was verified via PAGE (Fig. S2).

### Expression and purification of HMPV N K171A/R186A.

The CAN97-83 HMPV N mutant was generated using QuikChange site-directed mutagenesis in pUC57 and subcloned into pET29b(+) using BamHI and XbaI cleavage sites. The construct was expressed, induced, lysed, and spun as described for N^0^-P. The crude lysate was loaded onto a column containing resin as described for N-RNA, and the resin was washed once with 20 mM Tris, 500 mM NaCl, and 10 mM imidazole (pH 7) and once with 20 mM Tris and 500 mM NaCl (pH 7). The protein was eluted with 20 mM Tris, 500 mM NaCl, and 300 mM imidazole (pH 7). The eluate was concentrated, and the NaCl concentration of the sample was adjusted to 100 mM using 20 mM Tris (pH 7). Then, the sample was heparin purified as described for N-RNA. Fractions were buffer exchanged and stored as described above. Protein quality was verified via PAGE (Fig. S2).

### Expression and purification of RSV A2 P.

RSV A2 P constructs were expressed as MBP-His tag fusion proteins in BL21(DE3) E. coli cells (Novagen) using a modified pET15b vector. At an optical density at 600 nm (OD_600_) of 0.6 to 0.7, recombinant protein expression was induced with 0.5 mM isopropyl-β-d-1-thiogalactopyranoside (IPTG) for 12 to 14 h at 18°C. Cells were harvested and resuspended in lysis buffer containing 20 mM Tris (pH 7.5), 150 mM NaCl, 20 mM imidazole, and 5 mM 2-mecaptoethanol (BME) with protease inhibitors. Cells were lysed using an EmulsiFlex-C5 homogenizer (Avestin), and lysates were clarified by centrifugation at 30,000 × *g* at 4°C for 40 min. P proteins were purified sequentially using affinity tag columns (Ni and amylose), ion exchange Q column, and gel filtration column (10 mM HEPES, 150 mM NaCl, and 2 mM tris(2-carboxyethyl)phosphine hydrochloride [TCEP]). Fractions were pooled and concentrated and then aliquoted and flash frozen in liquid nitrogen. The purity of P proteins was determined by Coomassie staining of SDS-PAGE. The MBP-His tag was removed by the TEV protease (TEV) protease. To confirm protein quality, at the time of droplet assays, a side-by-side PAGE was run with HMPV P (anion) and RSV P (Fig. S2).

### Protein labeling.

Prior to buffer exchange, purified HMPV N^0^-P was labeled with Alexa 488 TFP ester (ThermoFisher). The Alexa 488 tetrafluorophenyl (TFP) ester was prepared with DMSO to make a 10-mg/mL solution. The solution was added dropwise to the protein sample. The sample rocked for 1 h in the dark and was buffer exchanged and stored as described above. Anion exchange-purified HMPV P was labeled in a similar manner using the Alexa 594 *N*-hydroxysuccinimide (NHS) ester (ThermoFisher).

### Droplet assay.

A 20% dextran solution was prepared in 25 mM HEPES and 150 mM KCl (protein storage buffer; pH 7.5). Dithiothreitol (DTT) was added to the dextran solution to give a final concentration of 1 mM. HMPV protein constructs were diluted in the 20% dextran, 1 mM DTT, 25 mM HEPES, and 150 mM KCl (pH 7.5) solution in 1.5-mL Eppendorf tubes. This solution was used in samples for standard droplet imaging, fusion droplet imaging, and turbidity assays. For the HMPV P samples tested at different KCl concentrations, similar buffers were prepared with KCl ranging from 0 mM to 500 mM. A total of 1.5 μL of sample was placed on an 8-well printed microscopy slide and covered with a glass coverslip. For droplets imaged at later time points, the slides were stored in a humidified chamber.

### Droplet microscopy imaging.

HMPV purified protein samples were imaged using either DIC or epifluorescence on a Nikon Eclipse E600 instrument with the 60× objective or on an Axiovert 200M instrument with a 100× oil objective. Fusion time lapse images were acquired with MetaMorph software using DIC on a Zeiss Axiovert 200M microscope with the 100× oil objective. Images were acquired at 0.3-sec or 0.5-sec intervals.

### RNA oligomer.

The fluorescent RNA decamer was purchased from Integrated DNA Technologies. It was terminated with OH at the 5′ end and 6-carboxyfluorescein at the 3′ end.

### Turbidity assay.

Protein solutions were mixed with 20% dextran, 1 mM DTT, 25 mM HEPES, and 150 mM KCl (pH 7.5) in clear 96-well plates. The final concentration of the protein was 40 μM. The absorbance of the solutions was measured on a SpectraMax iD3 instrument at 395 nm (20). Readings were taken at 5-min intervals for 8 h or longer.

### Live-cell imaging.

VeroE6 cells were seeded in 12-well glass-bottom culture plates and the day after were infected with HMPV-mCherryP using an MOI of 3. Cells were kept at 37°C in a 5% CO_2_ atmosphere until imaging. Images were acquired in a LionHeartFX fluorescence microscope using a 60× oil immersion objective. At 24, 48, and 72 hpi, infected cultures were imaged for 10 min, with images taken every 30 s. At least 5 different infected cells were imaged per condition. Alternatively, Vero cells were electroporated with 100 ng of a plasmid encoding mCherryP using a Neon system (ThermoFisher), pulsed at 220V and 950 μF, and subsequently seeded in 6-well glass bottom culture plates. Twenty-four hours postelectroporation, cells were infected with rgHMPV at an MOI of 3 and kept for another 48 h at 37°C in a 5% CO_2_ atmosphere. Cells were imaged in a NikonA1 confocal microscope, acquiring images every 25 s using the 60× oil immersion objective.
