# Supplementary figures and images for: Human Metapneumovirus Phosphoprotein Independently Drives Phase Separation and Recruits Nucleoprotein to Liquid-Like Bodies
Source: mBio. 2022 May 10;13(3):e01099-22. doi: 10.1128/mbio.01099-22 (PMC9239117; doi:10.1128/mbio.01099-22)

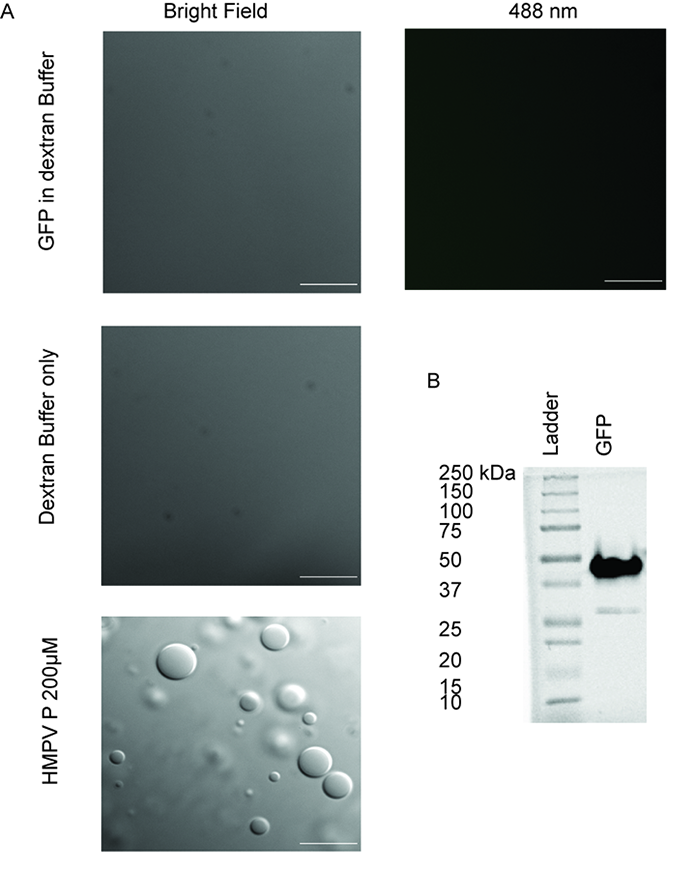

Supplement: FIG S3 [file mbio.01099-22-s0003.tif]

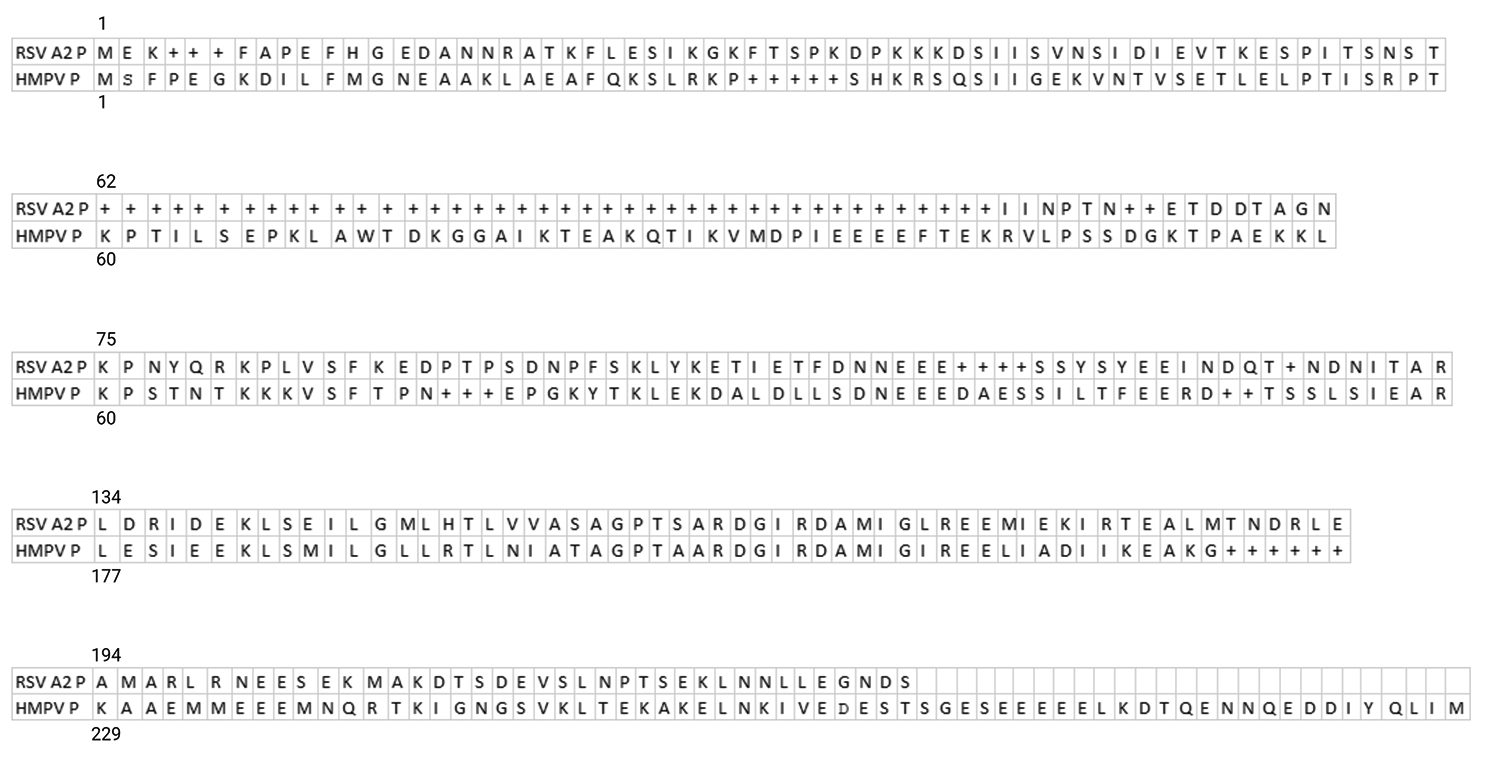

Supplement: FIG S4 [file mbio.01099-22-s0004.tif]

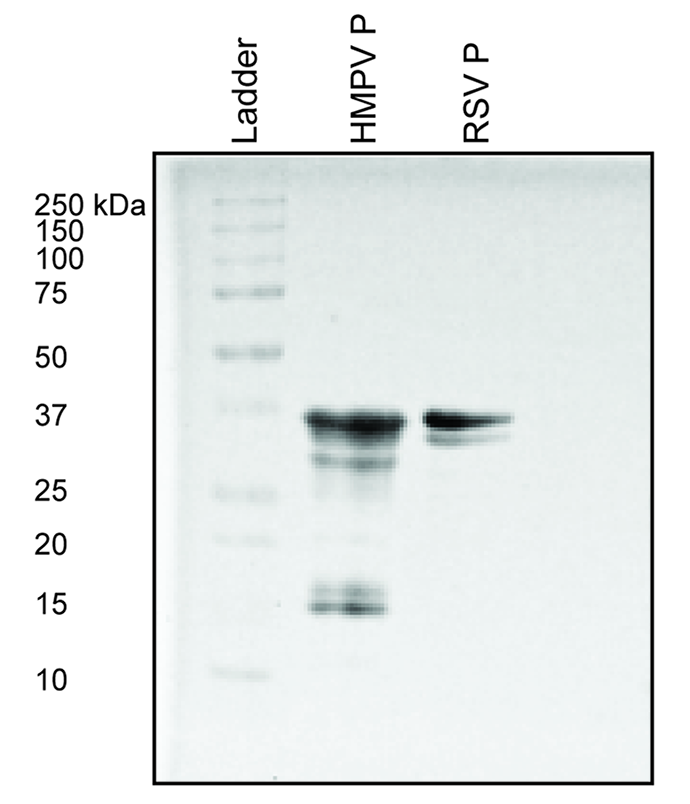

Supplement: FIG S2 [file mbio.01099-22-s0002.tif]

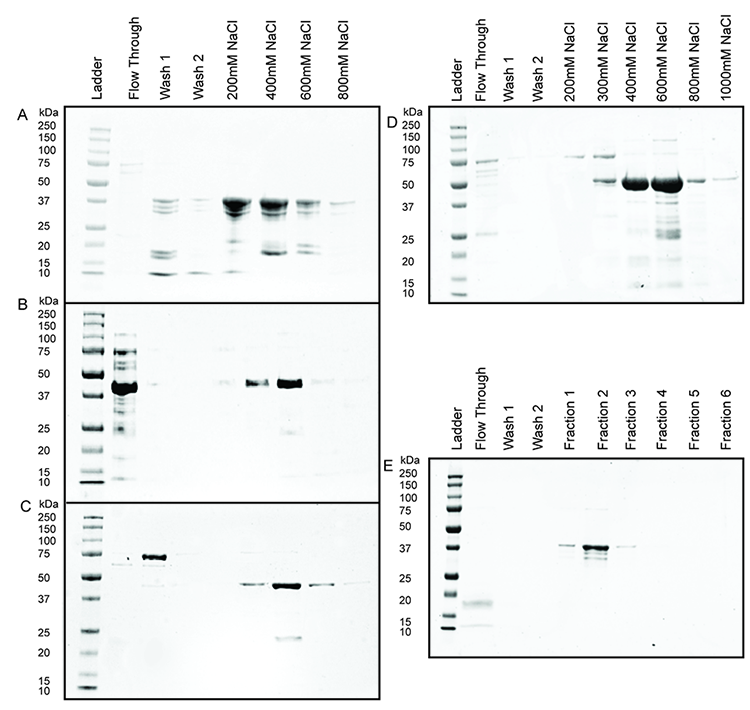

Supplement: FIG S1 [file mbio.01099-22-s0001.tif]
